# Biomarkers for Post-Traumatic Headache in Youth

**DOI:** 10.1007/s11916-026-01571-8

**Published:** 2026-10-30

**Authors:** Carlyn Patterson Gentile, Ramon Diaz-Arrastia

**Affiliations:** 1 Department of Pediatrics, Children’s Hospital of Philadelphia, 3401 Civic Center Blvd, Philadelphia, PA 19104 USA; 2 Department of Neurology, University of Pennsylvania, 3400 Spruce Street, Philadelphia, PA 19104 USA

**Keywords:** Post-traumatic headache, Traumatic brain injury, Biomarkers, Pediatric

## Abstract

**Purpose of Review:**

Traumatic brain injury is a highly prevalent condition impacting youth that commonly results in persist headache. However, current management is limited by our ability to differentiate youth who will recover with little intervention from those who will develop a potentially debilitating headache lasting months to years. The aim of this review is to highlight promising biomarkers for identifying youth who will develop persistent post-traumatic headache.

**Recent Findings:**

Multiple candidate genetic, protein, imaging, electrophysiological, and digital biomarkers have been identified to help differentiate youth who will develop persistent headache from traumatic brain injury from those who will recover. Promising directions include biomarkers that overlap between traumatic brain injury and migraine. However, no biomarkers are ready for standard use in clinical care.

**Summary:**

While further work is needed to develop and validate clinically reliable biomarkers of post-traumatic headache in the pediatric population, myriad candidate biomarkers hold promise and warrant further study.

## Introduction

Traumatic brain injury (TBI) is highly prevalent, impacting 1.8 million youth (2.5%) in the United States [1]. While most will recover within 2–4 weeks, over 30% will experience persistent symptoms that last longer than 1 month [2]. Headache is the most common symptom; over 80% of youth will report headache within the first 48 h of injury [3], ~ 25% will continue to have headache that lasts longer than 1 month [3], and ~ 8% will report headache that lasts longer than 3 months meeting the International Classification of Headache Disorders (ICHD-3) definition of persistent post-traumatic headache (PTH) [4, 5]. One of the greatest challenges in TBI management is distinguishing those who will recover quickly with little-to-no intervention from those who will develop persistent symptoms, including headache. This is critical to providing headache-directed care early to those who need it [6]. Primarily, clinicians have relied on factors associated with prolonged recovery and PTH including female sex [7], prior history of concussion [7], high acute post-injury symptom burden [8], post-traumatic migraine-like symptoms [9], and family or personal history of migraine [10]. The presence of headache has factored prominently in the validated 5P clinical risk score for predicting persistent symptoms in youth with acute concussion [11]. While this is a good start, it is insufficient.

Biomarkers (i.e., indicators of normal or pathologic biological processes) offer great promise to identify youth at risk for prolonged PTH and guide tailored treatment that aims to change the trajectory of recovery. Indeed, they have featured prominently in the Clinical, Biomarker, Imaging, and Modifiers (CBI-M) framework proposed to reclassify TBI to more accurately reflect long-term outcomes [12]. CBI-M was developed in response to the National Academies of Sciences, Engineering, and Medicine (NASEM) report in 2022, which made foundational recommendations to redefine TBI classification [13]. For the purposes of this review, we will refer to brain injury in the terms used by the original studies, with the recognition that CBI-M-based nosology will likely replace terms like mild TBI (mTBI) and concussion in the coming years.

**Fig. 1:**
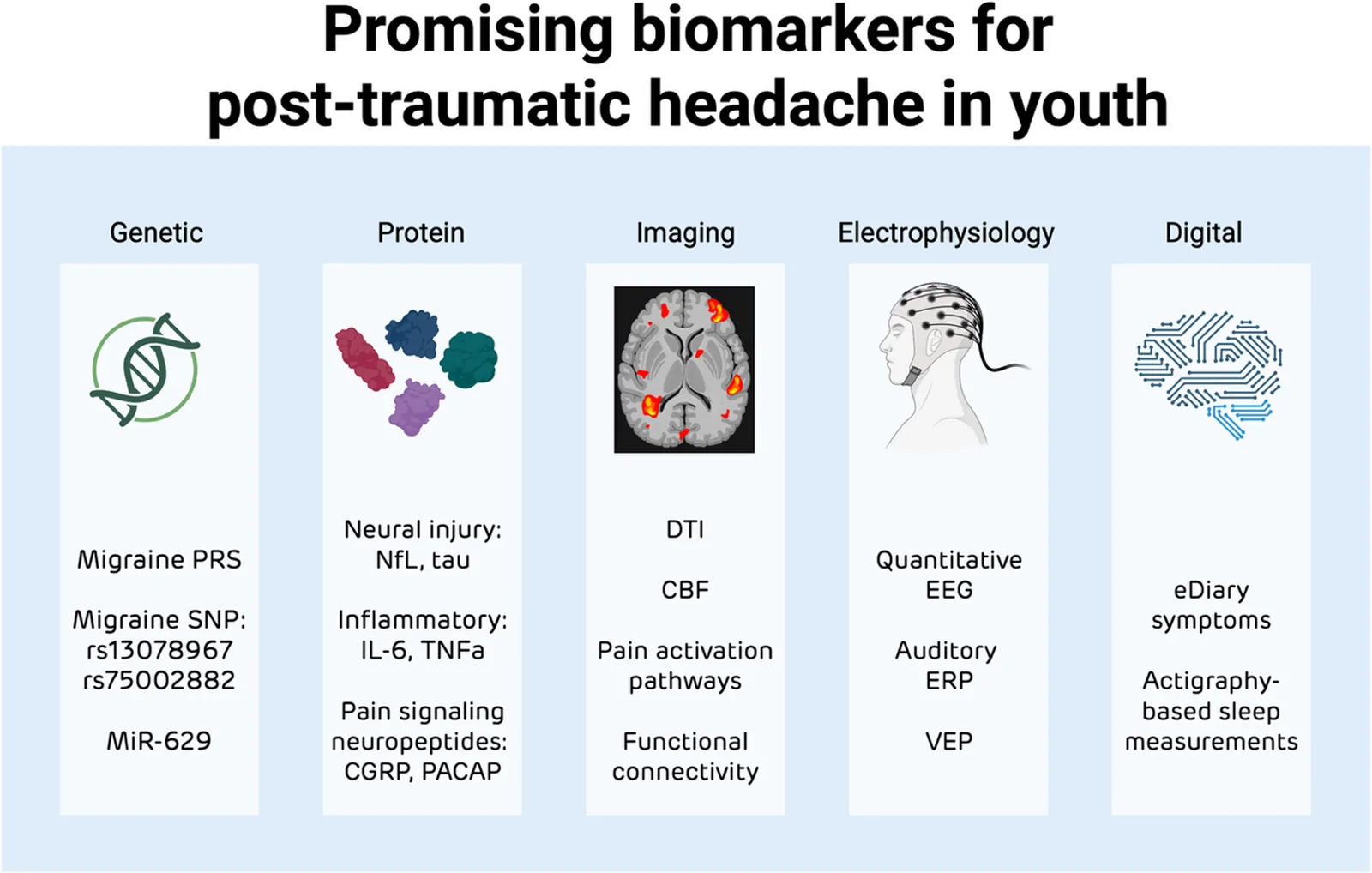
Candidate biomarkers for post-traumatic headache. Created in BioRender. Patterson Gentile, C. (2026) https://BioRender.com/cs2jvpe

Biomarkers serve different purposes, including identifying susceptibility and risk for prolonged recovery and persistent PTH, predicting long-term outcomes, and predicting or monitoring for response to a therapy. All serve the goal of providing more targeted, personalized care to define risk of prolonged recovery and improve outcomes, and ultimately prevent the development prolonged PTH. The focus of this review is on the pediatric population, which is a critical given the high and increasing prevalence of TBI in this population [14]. The pathophysiology of TBI likely overlaps substantially in pediatric and adult populations, thus adult studies have been included in this review. However, the interaction between brain development and TBI creates added challenges that uniquely face biomarker development in pediatric populations, which are discussed. While no biomarkers of TBI or PTH have been approved for clinical use in the pediatric population and due to current limited clinical utility [15, 16], there are multiple promising biomarker modalities that will be covered in this review (see Fig. 1, and Table 1 for summaries). Table 1 includes a qualitative appraisal on strength of findings based on the authors assessment, with a summary of findings and limitations for each candidate biomarker. Given that PTH biomarker development is an emerging field, promising biomarkers within TBI and their possible application to PTH specifically will also be reviewed. All reported studies and experiments with human or animal subjects performed by the authors have been previously published and complied with all applicable ethical standards (including the Helsinki declaration and its amendments, institutional/national research committee standards, and international/national/institutional guidelines).

**Table 1:** Candidate biomarkers for post-traumatic headache in youth

	Biomarker	Type of biomarker / potential use	Population studied	Outcome	Strength of evidence**
Markers of Neural Injury	GFAP, UCH-L1	Acute TBI diagnostic biomarker	Primarily adults, some pediatric data	Acute TBI, not predictive of persistent PTH or PPCS	Strong for acute TBI detection/severity; negative/limited for prognosis of PPCS or PTH. FDA approved to determine the need for head CT in acute head injury.
	S100B	Acute TBI diagnostic biomarker	Primarily adults, some pediatric data	Acute TBI, not predictive of persistent PTH or PPCS	Moderate for acute TBI/CT triage; negative for prolonged recovery or PTH prediction.
	NfL	Prognostic marker for prolonged recovery	Primarily adults, some pediatric data	PPCS, not PTH-specific	Moderate for prolonged recovery/PPCS, unknown for PTH.
	Total tau, p-tau181, p-tau231, and p-tau/total tau ratio	Diagnostic, prognostic biomarker	Primarily adults, some adolescent data	Acute TBI and PPCS/general outcomes, not PTH-specific	Preliminary for acute TBI severity, unknown for PTH.
	NSE	Diagnostic, prognostic biomarker	Primarily adults, some pediatric data	PPCS and headache measures, not useful for PTH based on current evidence	Negative/limited.
	Multimarker neural-injury panels (e.g., GFAP, UCH-L1, NfL, S100B, NSE, tau)	Diagnostic, prognostic biomarker	Adults	General TBI outcome, not PTH-specific	Moderate for long-term TBI outcomes, limited for PTH.
Markers of inflammation	IL-6	Prognostic biomarker	Adults and children	PPCS/general TBI recovery, no direct PTH studies	Moderate for TBI and prolonged recovery. It is also implicated in migraine, supporting biologic plausibility for PTH.
	TNF-$\:\alpha\:$	Prognostic and treatment-response biomarker	Primarily children for PPCS; migraine literature includes children and adults	PPCS, no direct PTH prognostic or treatment-response studies	Preliminary to moderate. Associated with worse outcomes after mTBI and elevated in children who develop PPCS. Migraine studies suggest levels may decrease with effective preventive treatment, but this has not been evaluated in PTH.
	MCP-1/CCL2, IL-1$\:\beta\:$, IL-1RA, and IL-10	Prognostic biomarker panel	Primarily adults	TBI outcomes, not PTH-specific	Preliminary. Meta-analysis supports elevations after mTBI and associations of several cytokines with worse clinical outcomes, but PTH-specific data are absent.
	Salivary miR-629	Prognostic biomarker	Children and young adults	PTH at 4 weeks	Preliminary but PTH-specific.
Migraine Susceptibility	Migraine-related SNPs rs13078967 and rs75002882	Susceptibility, risk biomarker	Children	Post-traumatic migraine-like headache/PTH	Preliminary, PTH-specific.
	Migraine polygenic risk score	Susceptibility, risk biomarker	Children	PTH/ migraine-like PTH	Negative/limited. Further PRS refinement and validation are needed.
	Personal or family history of migraine	Susceptibility, risk biomarker	Adult and pediatric	PPCS/prolonged recovery and PTH risk, PTH/ migraine-like PTH	Moderate, but mixed. Many studies associate migraine history with prolonged concussion recovery, although findings are not universal and family history may be underreported.
Migraine-related neuropeptide	CGRP	Diagnostic, predictive, and treatment-response biomarker	Adult PTH, pediatric acute TBI, animal models	Persistent PTH in adults; acute TBI in children;,treatment response remains untested	Strong biologic rationale, preliminary and inconsistent clinical biomarker evidence.
	PACAP	Diagnostic, predictive, and treatment-response biomarker	Adult PTH, pediatric acute TBI, animal models	Persistent PTH in adults, acute TBI in children, treatment response remains untested	Strong biologic rationale, preliminary/limited clinical biomarker evidence.
Structural Brain MRI	Cortical thickness/ gray matter density	Imaging marker to identify persistent PTH phenotype	Adults	Persistent PTH	Preliminary and inconsistent.
	DTI white-matter measures	Diagnostic and monitoring biomarker	Primarily adults for PTH; children for PPCS	Persistent PTH in adults, PPCS in children	Moderate for TBI-related white-matter injury; preliminary/inconsistent for PTH. Some studies identified corpus callosum and fornix-septo-hippocampal changes in PTH, while others found no PTH-specific differences.
	Quantitative MRI cortical microstructure	Diagnostic and monitoring biomarker	Adults	Persistent PTH	Preliminary. One study found occipital and temporal microstructural changes suggestive of neuroinflammation in persistent PTH.
	White matter hyperintensities and cerebral microbleeds	Diagnostic biomarker	Adults	Persistent PTH	Negative for persistent PTH. No differences were found between adults with persistent PTH and healthy controls.
	Lateral geniculate nucleus iron deposition	Prognostic biomarker	Adults	Acute PTH predicting 3-month headache severity	Preliminary but directly PTH-relevant. Acute iron deposition correlated with headache intensity at 3 months.
Functional Brain MRI	Periaqueductal gray–default mode network connectivity	Prognostic biomarker	Adults	Persistent PTH	Preliminary but directly PTH-specific. Disrupted connectivity predicted persistent PTH at 3 months.
	Cerebral blood flow/perfusion	Prognostic marker	Primarily children, some adult data	PPCS primarily, limited PTH-specific evidence	Mixed. Higher global CBF and declining CBF over time were associated with PPCS and favorable recovery in children, but CBF did not distinguish females with and without PTH at 2 weeks. Regional perfusion may better correlate with symptoms than global CBF.
	Cerebrovascular reactivity	Diagnostic and severity biomarker	Adults	Chronic PTH/headache severity	Preliminary. Associated with headache severity in chronic moderate-to-severe TBI.
	Pain-induced fMRI activation	Monitoring and treatment efficacy biomarker	Adults	Persistent PTH and headache improvement over time	Preliminary but promising and PTH specific. Persistent PTH was associated with sustained pain-related activation, whereas headache improvement was accompanied by normalization of activation.
	Resting-state functional connectivity	Diagnostic biomarker	Adults	Persistent PTH versus migraine	Preliminary. Functional connectivity differed between persistent PTH and migraine despite overlapping symptoms.
Electrophysiology	Quantitative EEG	Diagnostic and prognostic biomarker for TBI/prolonged recovery	Adult and pediatric	TBI diagnosis and PPCS, not PTH-specific	Preliminary to moderate for TBI classification and prolonged recovery, limited for PTH.
	Auditory P300 event-related potential	Electrophysiologic marker of PTH phenotype	Adults	PTH	Preliminary but PTH-specific. Delayed P300 response observed in PTH and correlated with anxiety and depression symptoms.
	Pain-related evoked potentials	Diagnostic biomarker	Adults	PTH	Preliminary but PTH-specific. Reduced N1P1 amplitudes were observed in adults with PTH.
	Visual evoked potentials/ visual cortical hyperexcitability	Electrophysiologic marker of migraine-like PTH phenotype	Adolescents and adults	Acute concussion with migraine-like symptoms; not persistent PTH-specific	Preliminary. Enhanced VEPs have been reported in acute post-traumatic photophobia and in adolescents with migraine-like symptoms after concussion; studies are small and preliminary.
Digital	Headache eDiary	Prognostic biomarker; recovery monitoring	Adults	Acute PTH predicting persistent severe headache	Preliminary but directly PTH-relevant.
	Actigraphy-based sleep metrics	Prognostic biomarker; modifiable risk biomarker	Pediatric	PPCS/general recovery; not PTH-specific	Preliminary and mixed.

*CBF* cerebral blood flow, *CGRP* calcitonin gene-related peptide, *CT* computed tomography, *DTI* diffusion tensor imaging, *EEG*   electroencephalography, *GFAP*  glial fibrillary acidic protein, *IL*  interleukin, *MCP-1/CCL2*  monocyte chemoattractant protein-1/C-C motif chemokine ligand 2, *NfL*  neurofilament light, *NSE*  neuron-specific enolase, *PACAP*  pituitary adenylate cyclase-activating polypeptide, *PPCS*  persistent post-concussive symptoms, *PTH*  post-traumatic headache, *SNP*  single nucleotide polymorphism, *TNF*-*α*  tumor necrosis factor-alpha, *UCH-L1*  ubiquitin C-terminal hydrolase L1, *VEP*  visual evoked potential

**Strength of evidence is a qualitative assessment based on authors’ evaluation of the included literature

## Biomarkers of Neural Injury

Not surprisingly, the most promising biomarkers to date have been inspired by TBI pathophysiology, including markers of neuronal, axonal, glial injury and neuroinflammation. Biomarkers measurable in accessible biological fluids such as blood have been extensively studied in acute TBI, and emerging research has also looked at their potential for predicting prolonged recovery [17]. Neuroimaging and electrophysiological markers have also been explored [18–20]. However, most studies have focused on adult populations, and studies indicate that these biomarkers are more effective at identifying TBI has occurred immediately after the injury or predicting prolonged recovery in general. The few studies that have looked at PTH as an outcome have largely been inconsistent.

### Protein Biomarkers of Brain Injury

Glial Fibrillary Acidic Protein (GFAP), Ubiquitin C-terminal Hydrolase L1 (UCH-L1), and the calcium binding protein S100b are blood biomarkers of neural injury with greatest utility in the acute TBI setting, rather than for identifying or predicting prolonged recovery or PTH. GFAP reflects astrocytic injury and rises within 30 minutes, peaks at approximately 20 hours, and declines over the following 3 days, although elevations may persist for up to 1 month [21–25]. UCH-L1, a marker of axonal integrity, peaks earlier at approximately 8 hours and is more transiently elevated, declining substantially within 48 hours [23, 26]. Combined GFAP and UCH-L1 testing is FDA-cleared in adults to help rule out clinically significant intracranial hemorrhage during the first 24 hours after injury; its high sensitivity but limited specificity makes it useful for determining whether CT imaging may be necessary [27, 28]. Pediatric data are less established, particularly because baseline GFAP is higher in prepubertal children [29]. In children, GFAP and UCH-L1 are elevated during post-injury days 0–3, while S100B may reduce unnecessary CT use in acute TBI [25, 30, 31]. Although GFAP has been associated with acute symptoms, vomiting, and poor short-term outcomes [32, 33], GFAP, UCH-L1, and S100B have not been found to predict persistent symptoms nor PTH specifically in adolescent nor adult population [17, 34, 35].

Neurofilament light (NfL) and Tau-based biomarkers that are important for axonal and neuronal integrity, respectively, have shown promise for predicting prolonged recovery, although their value for PTH specifically remains uncertain. NfL rises modestly within the first day after injury, peaks later at 2–4 weeks and can remain elevated for months in pediatric and adult populations [25, 36–39]. Higher NfL has been associated with longer return-to-play, persistent post-concussive symptoms (PPCS), post-traumatic amnesia, and loss of consciousness [24, 36, 38, 39]. However, not all findings have been consistent; one longitudinal adolescent sports-concussion study found no post-injury NfL increase compared with pre-injury values [40]. Blood p-tau231 can distinguish CT-positive from CT-negative TBI, while p-tau231 and p-tau231/total tau ratios have been associated with persistent symptoms at 6 and 12 months [41, 42]. Total tau has correlated with symptom burden during the first post-injury month in adolescents, and p-tau181 has been associated with subacute cognitive symptoms in youth [25, 43]. Brain-derived tau may predict long-term outcomes after severe TBI [44], but its relevance to mild TBI and PTH requires further study. In contrast, neuron-specific enolase (NSE) has not been associated with persistent symptoms or PTH measures in systematic reviews and large prospective cohorts [34, 45, 46]. 

Across biomarker studies, GFAP and UCH-L1 show the strongest associations with acute injury severity, hospitalization, and mortality, whereas NfL has the strongest association with 6-month outcomes [17]. Panels—including GFAP, UCH-L1, NfL, S100B, NSE, and tau—may provide incremental prognostic information for general 6-month TBI outcomes [47]. However, few studies directly compare individuals with and without PTH. Consequently, neural-injury biomarkers currently have limited utility for identifying or predicting PTH specifically. Their association with injury severity may also be poorly aligned with PTH risk, as headache is often more common after mild than severe TBI [48–52]. 

### Biomarkers of Inflammation

As TBI triggers a neuroinflammatory response, inflammatory cytokines have been biomarker targets. A systematic review and meta-analysis of 26 papers found that interleukin-6 (IL-6), monocyte chemoattractant protein-1/C-C motif chemokine ligand 2 (MCP-1/CCL2), and interleukin-1-beta (IL-1b) were elevated in mTBI compared to healthy controls. Additionally, the meta-analysis revealed that elevations of IL-6, tumor necrosis factor-alpha (TNF-a), IL-1RA, IL-10, and MCP-1/CCL2 were associated with worse clinical outcomes [53]. IL-6 is the most promising of inflammatory biomarkers. It is elevated within 6 hours of TBI [54, 55], is associated with symptom duration [55], and is elevated in the chronic stage after mTBI [56]. IL-6 and TNF-a have also been explored in their relationship to migraine (see section below). Measuring post-translational regulators of gene expression have also identified evidence of inflammation as a predictor of prolonged recovery, including headache. A study that measured salivary microRNA within 14 days of injury of 52 children and young adults found that miR-629, which regulates targets involved in inflammatory regulation, was specifically associated with headache at 4 weeks post-injury [57].

### Alterations in Brain Structure and Function

Evaluating structural and functional differences with MRI have been developed as diagnostic, prognostic, and monitoring biomarkers. Both gray and white matter differences in those with PTH have been identified by structural MRI, though results have been mixed and performed primarily in adults. Diffusion Tensor Imaging (DTI) studies consistently identify white-matter changes after TBI [18, 19], but PTH-specific findings are mixed. Some studies reported altered fractional anisotropy in the corpus callosum and fornix-septo-hippocampal circuit among individuals with PTH, whereas others found no DTI differences between PTH, migraine, or control groups [58–61]. Similarly, some studies found persistent PTH to be associated with reduced frontal and parietal cortical thickness, while other studies found no differences in gray-matter density [62–64]. Emerging quantitative MRI data suggest microstructural changes consistent with neuroinflammation in the occipital cortex and temporal gyrus in persistent PTH [65]. Conversely, white matter hyperintensities and cerebral microbleeds did not differ between those with persistent PTH and healthy controls [66]. Increased iron deposition in the lateral geniculate nucleus during acute PTH correlated with headache intensity at 3 months, suggesting potential prognostic utility [67]. Pediatric imaging studies have largely evaluated PPCS rather than PTH specifically. In the A-CAP study, DTI abnormalities suggestive of neuroinflammation and axonal swelling persisted up to 6 months after injury, particularly in younger children with persistent symptoms [68].

Functional MRI findings also support altered brain networks in PTH. Disrupted connectivity between the periaqueductal gray and default mode network predicted persistent PTH at 3 months [69]. Cerebral blood flow may have prognostic relevance for PPCS: children with PPCS had higher global CBF at approximately 6 weeks, and declining CBF over time was associated with favorable recovery [70, 71]. However, CBF did not differ between females with and without PTH at 2 weeks post-concussion, limiting its current utility as an acute diagnostic biomarker for PTH [72]. Regional perfusion changes, rather than global CBF, were associated with symptom burden in youth with concussion [73]. In adults with moderate-to-severe TBI, cerebrovascular reactivity was associated with chronic PTH [74]. 

Overall, neuroimaging biomarkers show some promise for diagnostic and prognostic biomarkers of PTH, but work remains largely preliminary. In addition to challenges related to scalability of specialized MRI that will need to be addressed, most studies were small, used heterogeneous imaging methods, and cannot reliably distinguish PTH-specific changes from broader effects of TBI or PPCS [18–20]. 

Electroencephalography (EEG) has been explored as diagnostic and prognostic TBI biomarkers, though very little has been done to evaluate PTH specifically. Resting state EEG abnormalities have been consistently reported in those with sport-related concussion, the affected cortical rhythms have varied across studies [75]. Some studies have used EEG to separate those with and without TBI and identify those at risk for prolonged recovery. A study of in a military population found relative alpha power, global beta-band interhemispheric coherence was decreased relative to uninjured controls. Machine learning techniques considering multiple variables were able to predict correct classification with an accuracy of 75% [76]. Quantitative EEG was also used in combination with neurocognitive tests and concussion assessment tools to differentiate youth and young adults who experienced prolonged recovery (14 days or longer) [77]. Sensory evoked potentials have also been studied. TBI is generally associated with slowed and attenuated sensory evoked potentials in pediatric and adult populations thought to be a result of white matter injury [78–83]. The only studies that have specifically evaluated PTH have looked at sensory evoked potential correlates to distinguish those from PTH from those without and have only been done in adults. In those with TBI, the P300 event-related potential in an acoustic odd ball paradigm was delayed only in those with PTH, which correlated with anxiety and depression symptoms [84]. Another study found that pain-related evoked potential N1P1 amplitudes were reduced in adults with PTH [85]. 

Across modalities, biomarkers of neural injury are most useful for identifying acute TBI severity and may help predict prolonged recovery. However, studies to date have not established a link between markers of neural injury and PTH diagnosis or persistence, which is necessary to target early headache-directed care. It remains to be seen if this is due to an absence of studies that focus on PTH, or if the presence or severity of neural injury is not predictive of PTH as an outcome measure. These biomarkers also do not currently predict treatment response or guide therapy. Additionally, pediatric evidence remains limited, and further study is needed.

## Migraine-Related Biomarkers

It has long been recognized that there is substantial symptom overlap between migraine and PTH, which may reflect overlapping pathophysiology [86]. Therefore, significant interest has gathered around exploring risk factors and neural pathways implicated in migraine as predictors of persistent PTH and as a means to guide treatment.

### Migraine Genetic Risk

Predisposition for migraine has been identified as a promising risk factor or susceptibility marker for PTH. Many, though not all, studies have indicated that a personal or family history of migraine is a risk factor for prolonged recovery from concussion [10]. To date though, one study has measured the association between genetic risk of migraine with developing PTH with or without migraine-like symptoms [87]. They used a multipronged approach that included assessing a migraine polygenic risk score (PRS) developed for migraine diagnosis, 40 single nucleotide polymorphisms (SNPs) associated with migraine, and family history of migraine. They identified 4 migraine-related SNPs that were associated with post-traumatic migraine-like headache, two of which survived strict statistical correction (rs13078967 and rs75002882). Family history of migraine was also associated with PTH in some, but not all, of their statistical models. However, PRS did not differ across PTH outcome. While further work is needed as PRSs for migraine require further validation, and family history of migraine may be underreported, this study is the first to directly demonstrate a link between migraine genetic risk and PTH.

### Neuropeptides Associated with Migraine

The discovery and subsequent drug development targeting neuropeptides that play key roles in pain signaling in migraine has offered a promising avenue for PTH biomarker development, not only to predict persistent PTH, but also to guide treatment and predict treatment response. Calcitonin gene-related peptide (CGRP) has garnered the most attention, for good reason. CGRP is released from trigeminal nerve terminals, promoting peripheral and central sensitization that underlies migraine attacks [88]. Its discovery led to CGRP-targeted monoclonal antibodies and gepants, which are effective for acute and preventive migraine treatment [89]. 

Blocking CGRP is also a biologically plausible treatment target for PTH. In adults with persistent PTH, CGRP infusion can trigger migraine-like headache, supporting activation of CGRP-dependent trigeminal pain pathways [90, 91]. In murine TBI models, early CGRP blockade prevented PTH-like behaviors, whereas delayed treatment was ineffective, suggesting that timing of this therapy may be critical [92, 93]. Indeed, delayed treatment initiation along with heterogeneity in PTH mechanisms may help explain why clinical trials of CGRP blocking medicines to treat PTH in adults have been partially effective at best, and negative at worst [94–96]. Finding elevations in CGRP soon after injury could therefore help identify individuals most likely to benefit from early CGRP-targeted therapy and support prevention of persistent PTH.

However, the value of CGRP as a biomarker remains uncertain. Accurately measuring blood levels of CGRP remains a challenge due to variability in the reliability of assays, instability of CGRP, and sex differences [97, 98]. Flash freezing and use of peptidase inhibitors in collection tubes have been recommended. However, even under optimal collection and storage conditions, CGRP levels can decrease over time, which has limited its utility as a biomarker for migraine and PTH. These measurement challenges may help explain why studies of CGRP levels in adults with PTH have yielded mixed results. One study found elevated plasma CGRP levels in 86 adults with persistent symptoms for at least 2 months, most were female and had migraine-like headache [99]. By contrast, CGRP was not associated with migraine-like headache, TBI status, or headache burden in a study of 264 soldiers, mostly men, returning from deployment [46]. Another study found CGRP plasma levels were lower in individuals with persistent PTH compared to age and sex-matched controls [100]. No pediatric studies have specifically focused on CGRP levels in PTH, but one pediatric study compared children with acute mTBI compared to uninjured controls. They found higher CGRP levels in children with TBI with CT-positive findings [101]. While there are multiple challenges that face CGRP as a biomarker, there is also great potential.

Another promising biomarker for PTH related to migraine pathophysiology is pituitary adenylate cyclase-activating polypeptide (PACAP). PACAP and CGRP have shared and independent functions in migraine pathophysiology [102]. Like CGRP, PACAP has been a target for migraine therapeutics [102], has been found to induce a migraine-like headache in individuals with PTH [103]. This has led to interest in PACAP as a PTH biomarker to predict treatment response. In a TBI mouse model, blocking both CGRP and PACAP was more effective at reducing PTH-related pain behaviors than blocking CGRP or PACAP alone [104]. PACAP elevations have been observed in children with acute mTBI compared to uninjured controls, were higher in those with CT-positive findings, and correlated with CGRP levels, indicating PACAP may also have a role as a diagnostic biomarker in TBI [101]. PACAP has been found to interact with other inflammatory cytokines in TBI, mediating the relationship between repeated head trauma and elevations in IL-6 [105]. Taken together, these findings suggest further study of the role of PACAP in PTH is warranted.

### Migraine and TBI Markers of Inflammation

IL-6 and TNF-a are particularly intriguing for PTH as both are elevated during a migraine attack, IL-6 is elevated in those with migraine even between attacks [106, 107], and decreases in IL-6 and TNF-a have been associated with decreased headache frequency after effective treatment in children [108]. IL-6 and TNF-a showed post-injury dependent elevations in children with acute concussion who developed PPCS (>1 month) compared to children who recovered [109]. No studies in children nor adults have evaluated the prognostic value of IL-6 and TNF-a in PTH, thus further study is needed.

### Activation of Pain Pathways

There is evidence of enhanced activation in pain processing pathways that corresponds PTH in adults. In a task-based longitudinal fMRI study, pain-induced neural activations were higher in the postcentral gyrus, superior temporal gyrus, and inferior parietal gyrus, and ventral striatum compared to controls; those whose headaches improved demonstrated progressive normalization of these activations, while those with persistent symptoms demonstrated sustained activation [110]. This finding supports the use of pain-induced neural activations as an evaluative biomarker for treatment efficacy. It is important to note though that MRI studies have identified unique signatures for PTH. Specifically, functional connectivity differed between persistent PTH and migraine, despite similar symptom profiles [111, 112]. In contrast, enhanced stimulus evoked responses of sensory pathways may be a shared feature between migraine and PTH. Visual cortical hyper-excitability, which is seen in migraine [113, 114], and has been associated with migraine-related photophobia and visual aura [115, 116], has also been observed in TBI. Enhanced visually evoked potentials (VEPs) have been observed in an individual with acute post-traumatic photophobia [117], and have been reported in adolescents with migraine-like symptoms following concussion [118]. Current neuroimaging and electrophysiology studies are generally limited by small sample sizes, and further study is needed.

Overall, migraine-related biomarkers offer a promising avenue for identifying youth at risk for persistent PTH and for developing targeted early treatments. Emerging evidence links migraine genetic susceptibility, CGRP and PACAP signaling, inflammatory mediators such as IL-6 and TNF-$\:\alpha\:$, and altered pain-processing networks to PTH risk or persistence. These markers may ultimately help distinguish CGRP- or PACAP-responsive PTH phenotypes and guide targeted preventive therapy soon after injury. However, current evidence is limited by inconsistent biomarker findings, measurement challenges, heterogeneous PTH populations, small sample sizes, and sparse pediatric data. Larger, prospective studies with standardized collection methods and PTH-specific outcomes are needed before migraine-related biomarkers can be incorporated into clinical care.

## Digital Biometrics to Track Recovery

Digital metrics are an emerging area of biomarker development that provide unique tools to monitor recovery, predict long term outcomes, and identify modifiable risk factors for prolonged recovery and persistent PTH. Digital biomarkers include any digitally based metric from eDiary symptom trackers to continuous biometric data including sleep, physical activity, and heart rate from wearable biosensors. Digital devices, including personal cell phones, can be used to collect ecological momentary assessments that capture symptom evolution in near-real-time. In a study of 73 adults with PTH who were less than 60 days post-injury, using headache frequency and severity data from a daily headache eDiary supported development of a machine learning model that differentiated those would develop persistent severe headache from who would recover from 2 to 4 weeks of diary entries [119]. This approach has not yet been explored in youth with PTH. Biometrics from wearable biosensors is another direction in digital biomarker development. In 78 concussed youth, actigraphy-based sleep measurements within the first week post-injury helped predict recovery; those who slept too little or too much and those who took daytime naps within the first week of concussion recovered more slowly [120]. Another study did not find significant differences in commercially available actigraphy-based sleep measurements between concussed youth and uninjured controls [121]. While in its infancy, actigraphy-based measurements that can capture sleep and physical activity have great potential to identify modifiable risk factors of PTH.

## Challenges

While a lot of exciting work has been done in the field of biomarker development for TBI, and PTH more specifically, there are also barriers that are limiting the development of prognostic biomarkers for PTH in the pediatric population that should be considered.

### Defining PTH Persistence

There is a lack of consensus in the definition of “persistent symptoms” [122], which limits the ability to develop meaningful biomarkers. In the studies reviewed, persistent symptoms, including PTH, were defined from time periods ranging from two weeks to several months post-injury. As described above, multiple candidate biomarkers change in relation to time passed since injury, and symptoms present at the time of the evaluation. Biomarkers must be considered in the context of duration that has passed since injury, and the field of biomarker development would benefit from a more consistent definition of “persistent symptoms.”

### The Multifactorial Nature of PTH

TBI generally, and PTH specifically, are heterogenous conditions with multiple possible presentations, overlapping contributors, and potential endophenotypes [123, 124]. Factors that may contribute to PTH range from autonomic dysfunction, vestibular and oculomotor dysfunction, musculoskeletal injury and whiplash, and trauma-triggered activation of pain pathways common to migraine [86, 125–127]. Biomarkers have the potential to provide objective biological changes to help sort out the heterogeneity of PTH to develop tailored treatment. However, this also poses a challenge because finding meaningful biomarkers may necessitate large numbers with deep phenotyping to adequately address this heterogeneity.

### The Interaction Between Brain Injury and Development

An added challenge in developing biomarkers for the pediatric population is understanding how neurodevelopment interacts with TBI and PTH. There are multiple examples of how candidate biomarkers for PTH change as a function of age and developmental stage. Normative values of NF-L and GFAP display a U-shaped curve across age, with higher levels in infants and older adults, and a plateau in adolescence and early adulthood [128]. An fMRI study assessing functional connectivity in concussed youth found age-dependent between-network connectively differences that emerged 3-6 months following injury, after most clinically recovered [129]. Furthermore, there are developmental changes in electrophysiological measurements like VEPs [130]. Thus, definitions of normative values and abnormal cut-offs must take this age- and development-related changes into account.

## Conclusions

Biomarker development is a critical area of PTH research because it offers a means to provide early, targeted care for a condition that is inherently heterogeneous. To date, most studies have focused on prolonged recovery from TBI. Further study that focuses on PTH as a primary outcome measure is needed to help identify those who would benefit from headache-directed care to prevent protracted recovery and persistent PTH. 

## Key References

- Manley, G.T., et al., A new characterisation of acute traumatic brain injury: the NIH-NINDS TBI Classification and Nomenclature Initiative. Lancet Neurol, 2025. 24(6): p. 512–523.
  - ○ In response to the NASEM report, this paper outlines the new classification system proposed for acute TBI that better reflects long-term prognosis. This classification system consists of four pillars: clinical, biomarkers, imaging, and modifiers. Biomarkers factor prominently in this new classification system, highlighting their importance in predicting long-term outcomes from TBI, including persistent PTH.
- Davis, G.A., et al., Pediatric Sport-related Concussion: Recommendations From the Amsterdam Consensus Statement 2023. Pediatrics, 2024. 153(1): p. e2023063489.
  - ○ Current consensus statement recommendations that identify fluid and imaging biomarkers are of limited clinical utility in diagnosis pediatric sports-related concussion or predicting recovery.

## Data Availability

No datasets were generated or analysed during the current study.

## Abbreviations

CBF: Cerebral Blood Flow
CBI-M: Clinical, Biomarkers, Imaging, Modifiers
CGRP: Calcitonin Gene-Related Peptide
CT: Computed Tomography
DTI: Diffusion Tensor Imaging
EEG: Electroencephalography
FDA: Food and Drug Administration
GCS: Glasgow Coma Scale
GFAP: Glial Fibrillary Acidic Protein
IL: Interleukin
LOC: Loss of Consciousness
mAb: Monoclonal Antibody
MCP-1/CCL2: Monocyte Chemoattractant Protein-1/C-C Motif Chemokine Ligand 2
MRI: Magnetic Resonance Imaging
mTBI: Mild Traumatic Brain Injury
NASEM: National Academies of Sciences, Engineering, and Medicine
NfL: Neurofilament Light
NIH/NINDS: National Institutes of Health/National Institute of Neurological Disorders and Stroke
NSE: Neuron-Specific Enolase
PACAP: Pituitary Adenylate Cyclase-Activating Polypeptide
PPCS: Persistent Post-Concussive Symptoms
PRS: Polygenic Risk Score
PTA: Post-Traumatic Amnesia
PTH: Post-Traumatic Headache
TBI: Traumatic Brain Injury
TNFa: Tumor necrosis Factor-alpha
UCH-L1: Ubiquitin C-terminal Hydrolase L1
VEP: Visually Evoked Potential
